# Association of metformin, aspirin, and cancer incidence with mortality risk in adults with diabetes

**DOI:** 10.1093/jncics/pkad017

**Published:** 2023-03-01

**Authors:** Suzanne G Orchard, Jessica E Lockery, Jonathan C Broder, Michael E Ernst, Sara Espinoza, Peter Gibbs, Rory Wolfe, Galina Polekhina, Sophia Zoungas, Holli A Loomans-Kropp, Robyn L Woods, ASPREE Investigator Group, John McNeil, Robyn Woods, Rory Wolfe, Anne Murray, Andrew Chan, Suzanne Orchard, Jessica Lockery, Mark Nelson, Christorpher Reid, Raj Shah, Anne Newmann, Elsdon Storey, Nigel Stocks, Andrew Tonkin, Sara Espinoza

**Affiliations:** School of Public Health and Preventive Medicine, Monash University, Melbourne,VIC, Australia; School of Public Health and Preventive Medicine, Monash University, Melbourne,VIC, Australia; Translational Immunology and Nanotechnology Research Theme, School of Health and Biomedical Sciences, RMIT University, Bundoora, VIC, Australia; Department of Internal Medicine, Division of Cancer Prevention and Control, Ohio State University, Columbus, OH, USA; School of Public Health and Preventive Medicine, Monash University, Melbourne,VIC, Australia; Department of Pharmacy Practice and Science, College of Pharmacy and Department of Family Medicine, Carver College of Medicine, The University of Iowa, Iowa City, IA, USA; Division of Geriatrics, Gerontology and Palliative Medicine, Barshop Institute for Longevity and Aging Studies, University of Texas Health Science Center, and Geriatrics Research, Education and Clinical Center, South Texas Veterans Health Care System, San Antonio, TX, USA; The Walter & Eliza Hall Institute of Medical Research, Royal Parade, Parkville, Melbourne, VIC, Australia; Department of Medical Oncology, Peter MacCallum Cancer Centre, Melbourne, VIC, Australia; School of Public Health and Preventive Medicine, Monash University, Melbourne,VIC, Australia; School of Public Health and Preventive Medicine, Monash University, Melbourne,VIC, Australia; School of Public Health and Preventive Medicine, Monash University, Melbourne,VIC, Australia; Department of Internal Medicine, Division of Cancer Prevention and Control, Ohio State University, Columbus, OH, USA; Cancer Prevention Fellowship Program, Division of Cancer Prevention, National Cancer Institute, Rockville, MD, USA; School of Public Health and Preventive Medicine, Monash University, Melbourne,VIC, Australia

## Abstract

**Background:**

Metformin and aspirin are commonly co-prescribed to people with diabetes. Metformin may prevent cancer, but in older people (over 70 years), aspirin has been found to increase cancer mortality. This study examined whether metformin reduces cancer mortality and incidence in older people with diabetes; it used randomization to 100 mg aspirin or placebo in the ASPirin in Reducing Events in the Elderly (ASPREE) trial to quantify aspirin’s impact on metformin users.

**Methods:**

Analysis included community-dwelling ASPREE participants (aged ≥70 years, or ≥65 years for members of US minority populations) with diabetes. Diabetes was defined as a fasting blood glucose level greater than 125 mg/dL, self-report of diabetes, or antidiabetic medication use. Cox proportional hazards regression models were used to analyze the association of metformin and a metformin-aspirin interaction with cancer incidence and mortality, with adjustment for confounders.

**Results:**

Of 2045 participants with diabetes at enrollment, 965 were concurrently using metformin. Metformin was associated with a reduced cancer incidence risk (adjusted hazard ratio [HR] = 0.68, 95% confidence interval [CI] = 0.51 to 0.90), but no conclusive benefit for cancer mortality (adjusted HR = 0.72, 95% CI = 0.43 to 1.19). Metformin users randomized to aspirin had greater risk of cancer mortality compared with placebo (HR = 2.53, 95% CI = 1.18 to 5.43), but no effect was seen for cancer incidence (HR = 1.11, 95% CI = 0.75 to 1.64). The possible effect modification of aspirin on cancer mortality, however, was not statistically significant (interaction *P* = .11).

**Conclusions:**

In community-dwelling older adults with diabetes, metformin use was associated with reduced cancer incidence. Increased cancer mortality risk in metformin users randomized to aspirin warrants further investigation.

**ASPREE Trial Registration:**

ClinicalTrials.gov ID NCT01038583

Cancer is a leading cause of death worldwide (1,2). Cancer incidence is expected to increase in the next decade, with older people (eg, those aged over 70 years) at higher risk of incident cancer and cancer mortality (3). In the context of an aging population (4), prevention and treatment of cancer are a public health imperative.

Type 2 diabetes is a complex disease characterized by β-cell failure in the setting of insulin resistance (5); it is a known risk factor for several types of cancer, including liver, pancreatic, colorectal, breast, endometrial, and kidney cancer (6,7). In type 2 diabetes, systemic insulin resistance results in adaptive increases in β-cell mass and function, which initially conserve glucose homeostasis at the expense of elevated insulin levels. When this compensatory mechanism fails, hyperglycemia occurs (5). Thus, in most people with type 2 diabetes, hyperglycemia is associated with endogenous hyperinsulinemia. Although the underlying mechanism behind type 2 diabetes and cancer risk remains unclear, both hyperglycemia and hyperinsulinemia are associated with increases in the prevalence and mortality of malignancies (6,8-11), and both contribute to carcinogenic processes, including enhanced cellular proliferation, invasion, and apoptosis inhibition (12-14).

Metformin, an oral antihyperglycemic agent, is the recommended first-line treatment for type 2 diabetes in the absence of contraindications (12). Metformin acts by suppressing hepatic glucose production and increasing peripheral glucose uptake (13), thereby lowering blood glucose levels without increasing circulating insulin (14). This specific trait differentiates metformin from other antihyperglycemic medications, such as sulfonylureas and insulin therapy, which lower blood glucose levels by increasing plasma insulin concentrations (15). Recent analyses have suggested that their use may be associated with increased risk of cancer (16-18). In contrast, several studies have shown that metformin may protect against the development and progression of a variety of malignancies (19-26). Other observational studies, however, have reported no association between metformin use and cancer incidence or outcome, with authors citing methodological biases as tending to exaggerate the benefit of metformin (27-32). The conflicting evidence suggests that metformin may exercise different effects on cancer at different anatomical sites or, alternatively, that analyses of the effect of metformin in clinical practice may be complicated by other factors, such as co-prescribed medications or residual confounding caused by comorbid conditions.

Aspirin is commonly co-prescribed with metformin for prevention of cardiovascular disease in people with diabetes (33). Recent meta-analyses have found that low-dose aspirin, taken regularly for 4 to 5 years, could reduce cancer incidence, risk of metastatic spread, and cancer mortality over the subsequent 10 or more years (34-36). That said, a recent clinical trial of aspirin in older adults, the ASPirin in Reducing Events in the Elderly (ASPREE) study, showed no effect of aspirin on cancer incidence but an increased risk of cancer-related death (37,38). Furthermore, the A Study of Cardiovascular Events in Diabetes (ASCEND) clinical trial found no evidence of reduction in gastrointestinal or other cancer incidences in people with diabetes who were randomized to aspirin vs placebo after 7 years of treatment and follow-up (39). Bearing this is mind, it is possible that these medications have opposing effects on cancer prevention, with aspirin increasing and metformin decreasing risk. Disentangling the effects of metformin and aspirin may assist in explaining the conflicting evidence about metformin and cancer.

In this analysis, we aimed to use the randomization of participants to aspirin or placebo in the ASPREE trial to examine in older adults with diabetes 1) the association between metformin and cancer incidence and mortality, 2) the effect of aspirin (vs placebo) in metformin users on cancer incidence and mortality, and 3) whether the effect of aspirin (vs placebo) differs between those who do and do not use metformin.

## Methods

### The ASPREE clinical trial

This ASPREE trial was a secondary, intention-to-treat analysis of ASPREE clinical trial data (ClinicalTrials.gov ID NCT01038583). The ASPREE study enrolled community-dwelling individuals 70 years of age or older (≥65 years of age for members of US minority groups) with no major cardiovascular disease in Australia and the United States. Preexisting cancer was not an exclusion if life expectancy was beyond 5 years [19% of participants had preexisting cancer (40)]. Details regarding trial methods, recruitment, and outcomes have been described previously (37,41-44). Briefly, 19 114 participants were randomly assigned to aspirin (100 mg) or matching placebo and followed for a median of 4.7 years. Demographic data, including sex, race, ethnicity, smoking status, alcohol use and previous aspirin use were collected by participant self-report. Race/ethnicity categories are Caucasian/White and other, where other includes Aboriginal/Torres Strait Islanders, American Indian, Asian, Black/African American, Hispanic/Latino or Native Hawaiian/Other Pacific Islander/Maori. Ethics committees at each participating center approved the trial, and all participants provided written informed consent before enrollment.

### Event data collection and adjudication

Cancer was defined as diagnosis of any new primary cancer, excluding nonmelanoma skin cancer, that had been histopathologically confirmed or clinically evident on imaging. Cancer mortality was defined as death where the primary cause was attributable to cancer. Participants completed a questionnaire designed to record new cancer events at 6-month intervals, and clinical records were searched annually for new cancer diagnoses. All in-trial event reports (cancer and death) triggered the collection of clinical evidentiary documentation (eg, histopathology, specialist letters, imaging, and death certificates) from hospitals, pathology services, and responsible physicians. These clinical documents were compiled into an event summary and presented to a committee of international clinical experts specializing in oncology, for adjudication. Where histopathological confirmation was not undertaken clinically (eg, in the setting of diffuse metastatic disease or patient refusal of surgical intervention of any kind), cancer cases were considered to reach the cancer endpoint only if strong clinical evidence of disease was present on imaging (computed tomography, positron emission tomography, magnetic resonance imaging, or bone scans showing clear primary or diffuse metastatic disease) or blood biomarkers. Alternatively, clinically documented treatment for metastatic disease was considered sufficient to confirm the cancer endpoint. If the results of imaging investigations were unclear, suspicious, or inconclusive imaging, the cancer case was not considered a cancer endpoint. Further details of the cancer and cause-of-death adjudication processes have been published elsewhere (37,38).

### Collection and coding of medications

The ASPREE study defined baseline medications as any medications prescribed by a physician (or any nonsteroidal anti-inflammatory drug) and taken regularly at the time of randomization. Baseline medication data were collected directly from ASPREE participants, who brought their medications to the enrollment visit that immediately preceded randomization. Medication data were cross-checked with the participant’s medical record (when available), then transcribed into the ASPREE data system (45) and coded according to the World Health Organization Anatomical Therapeutic Chemical coding system (46). Detailed methods for the coding process have been published elsewhere (47).

### Definitions

**Figure 1.**
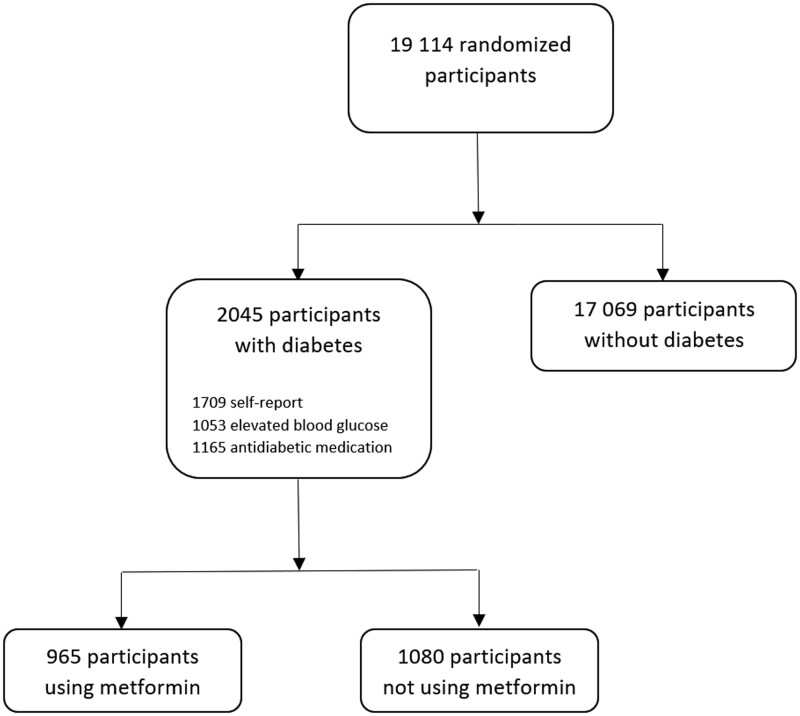
Cohort at baseline included in the current analysis. *Elevated blood glucose* refers to a fasting blood glucose level >125 mg/dL. Counts of participants with self-reported diabetes, elevated blood glucose, and anti-diabetic medication use are not mutually exclusive.

*Metformin use* refers to the prescription of a medication with an Anatomical Therapeutic Chemical code of A10BA02. Diabetes was defined as the presence at study entry of a high fasting blood glucose level (FBGL) (>125 mg/dL) (48), a self-report of diabetes, or prescription of an antihyperglycemic medication (see Supplementary Table 1, available online, for the full list). See Figure 1 for a flow diagram of the baseline cohort.

### Statistical analysis

The purpose of this secondary data analysis was to explore the long-term associations between metformin use and cancer based on the principles of intention to treat. Therefore, metformin exposure was defined as baseline metformin use only. A review of metformin use or nonuse over the follow-up period revealed that 83% of participants maintained consistency of either use or non-use of metformin. Cox proportional hazards regression models were used to analyze the relationship between metformin exposure at study baseline and cancer outcomes. For cancer incidence, the analysis was performed on the first cancer event (date of diagnosis) of any in-trial cancer, and censoring was defined at death if non–cancer-related death occurred (as a non–cancer-related death strongly indicated that cancer was not present) or the last date on which clinical event data were collected. For cancer mortality, the date of death was used as the event date, and censoring was defined at the end of the study, when the National Death Indices search was performed. Adjusted hazard ratios (HRs) were determined for incident cancer and mortality, which controlled for baseline factors identified as potential confounders. Because of limited sample size, cancer location site and stage were not analyzed. Competing-risks Nelson-Aalen cumulative incidence curves of cancer incidence and mortality are presented for participants with diabetes who do and do not use metformin.

To assess whether the association of metformin on outcome varied by therapeutic efficacy, additional Cox proportional hazard regression models included an interaction term between baseline blood glucose and metformin. Using these models, the log adjusted hazard ratio of metformin, across varying levels of blood glucose, were visualized using line plots.

The random allocation of ASPREE participants to aspirin or placebo was used to compare the aspirin effect between those who do and do not use metformin. Thus, these Cox proportional hazard regression models included an interaction between metformin and aspirin and were not adjusted for baseline factors.

Supplementary analysis was conducted using competing risks regression through Fine-Gray subdistribution hazard models. Deaths that occurred when participants were still at risk of cancer incidence were considered a competing risk of cancer incidence, but non–cancer-related deaths in participants with a cancer diagnosis were considered a competing risk of cancer mortality.

The proportional hazards assumption was assessed using tests of the Schoenfeld residuals against time (49), which showed that the assumption was satisfied in all models. Analyses, performed in R, version 4.0.2 (R Foundation for Statistical Computing), were 2-sided, with *P* < .05 considered statistically significant.

## Results

Of the 2045 participants with diabetes, 965 used metformin at baseline (median [Interquartile range, IQR] follow-up = 4.6 [3.5-5.5] years) and 1080 did not (median [IQR] follow-up = 4.5 [3.3-5.5] years) (Figure 1). Most participants with diabetes stayed within their baseline groups over follow-up (1698 of 2045 [83%]), although 107 (11%) participants using metformin at baseline stopped use during follow-up for at least 1 year, and 240 of 1080 (22%) participants not using metformin at baseline subsequently commenced metformin during follow-up. Table 1 shows baseline characteristics of ASPREE participants with diabetes, stratified by metformin use. Metformin users were more likely to be younger and not White, report previous regular aspirin use, have polypharmacy, have a body mass index of 25 kg/m^2^ or higher, and never have used alcohol compared with those who did not use metformin. Metformin users were also more likely to use other diabetes medications and have lower FBGLs than those not using metformin. Supplementary Table 2 (available online) shows baseline characteristics for participants who did not have diabetes.

**Table 1.** Baseline characteristics of ASPREE participants with diabetes^a^

	Diabetes			
Characteristic	Metformin (n = 965)	No metformin (n = 1080)	Total (N = 2045)	*P*
Age at randomization, No. (%)				
65-69 y	83 (9)	62 (6)	145 (7)	.009
70-74 y	496 (51)	541 (50)	1037 (51)	
75-79 y	255 (26)	288 (27)	543 (27)	
80-84 y	103 (11)	134 (12)	237 (12)	
≥85 y	28 (3)	55 (5)	83 (4)	
Sex, No. (%)				
Female				
Male	497 (52)	549 (51)	1046 (51)	.762
Ethnicity and race,^b^ No. (%)				
White/Caucasian	755 (78)	909 (84)	1664 (81)	<.001
Other				
BMI category, No. (%)				
≥25	854 (89)	917 (85)	1771 (87)	.032
Smoking status, No. (%)				
Current	47 (5)	51 (5)	98 (5)	.947
Former	424 (44)	482 (45)	906 (44)	
Never	494 (51)	547 (51)	1041 (51)	
Alcohol use, No. (%)				
Current	613 (64)	767 (71)	1380 (67)	<.001
Former	105 (11)	83 (8)	188 (9)	
Never	247 (26)	230 (21)	477 (23)	
Clinical features				
Previous regular aspirin use,^c^ No. (%)	190 (20)	169 (16)	359 (18)	.017
CKD,^d^ No. (%)	345 (38)	368 (36)	713 (37)	.385
Polypharmacy (≥5), No. (%)	641 (66)	424 (39)	1065 (52)	<.001
Personal cancer history, No. (%)	182 (19)	204 (19)	386 (19)	.971
Family cancer history,^e^ No. (%)	538 (56)	636 (59)	1174 (57)	.152
Physical component summary score,^f^ median (IQR)^g^	47.4 (39.4-53.5)	47.5 (39.8-54.1)	47.4 (39.7-53.7)	.671
Randomized treatment group, No. (%)				
Aspirin	516 (53)	508 (47)	1024 (50)	–
Placebo	449 (47)	572 (53)	1021 (50)	–
FBGL				
FBGL, mean (SD), mg/dL	132.8 (37.4)	129.5 (34.9)	131.0 (36.1)	.042
FBGL, mean (SD), mmol/L	7.4 (2.1)	7.2 (1.9)	7.3 (2.0)	–
Diabetes treatment, No. (%)				
Insulin	83 (9)	74 (7)	157 (8)	.138
Other antihyperglycemic medication use	364 (38)	137 (13)	501 (24)	<.001
Diabetes self-report, No. (%)				
Self-report diabetes only	–	413 (38)	413 (20)	–

a Missing data in total cohort (N = 19 114): age at randomization, n = 0; sex, n = 0; ethnicity, n = 0; BMI, n = 89; smoking, n = 0; alcohol use, n = 0; previous regular aspirin use, n = 2; CKD, n = 1350; polypharmacy, n = 0; family cancer history, n = 0; randomized treatment group, n = 0; physical component score of the SF-12, n = 8; personal cancer history, n = 22. Percentages exclude missing values from denominator. Baseline characteristics of participants without diabetes are shown in Supplementary Table 2 (available online). ASPREE = ASPirin Reducing Events in the Elderly; BMI = body mass index; CKD = chronic kidney disease; FBGL = fasting blood glucose level; SF-12 = 12-Item Short Form Survey.

b Ethnicity and race were collected through participant self-report: White/Caucasian or other; “other” consists of Aboriginal/Torres Strait Islanders, American Indian, Asian, Black/African American, Hispanic/Latino or Native Hawaiian/Other Pacific Islander/Maori.

c Previous regular aspirin use: self-reported regular aspirin use before entering the study.

d Stage III-V CKD: urine albumin-to- creatinine ratio ≥3 mg/mmol or estimated glomerular filtration rate <60 mL/min/1.73 m^2^.

e Family cancer history: cancer history in the participant’s blood relatives (mother, father, siblings, and children) self-reported at baseline; ovarian and cervical cancer history were included, which were not included in recent ASPREE publication (26).

f Physical component score: physical component score of the SF-12 quality-of-life questionnaire (61).

g IQR; Interquartile Range.

**Figure 2.**
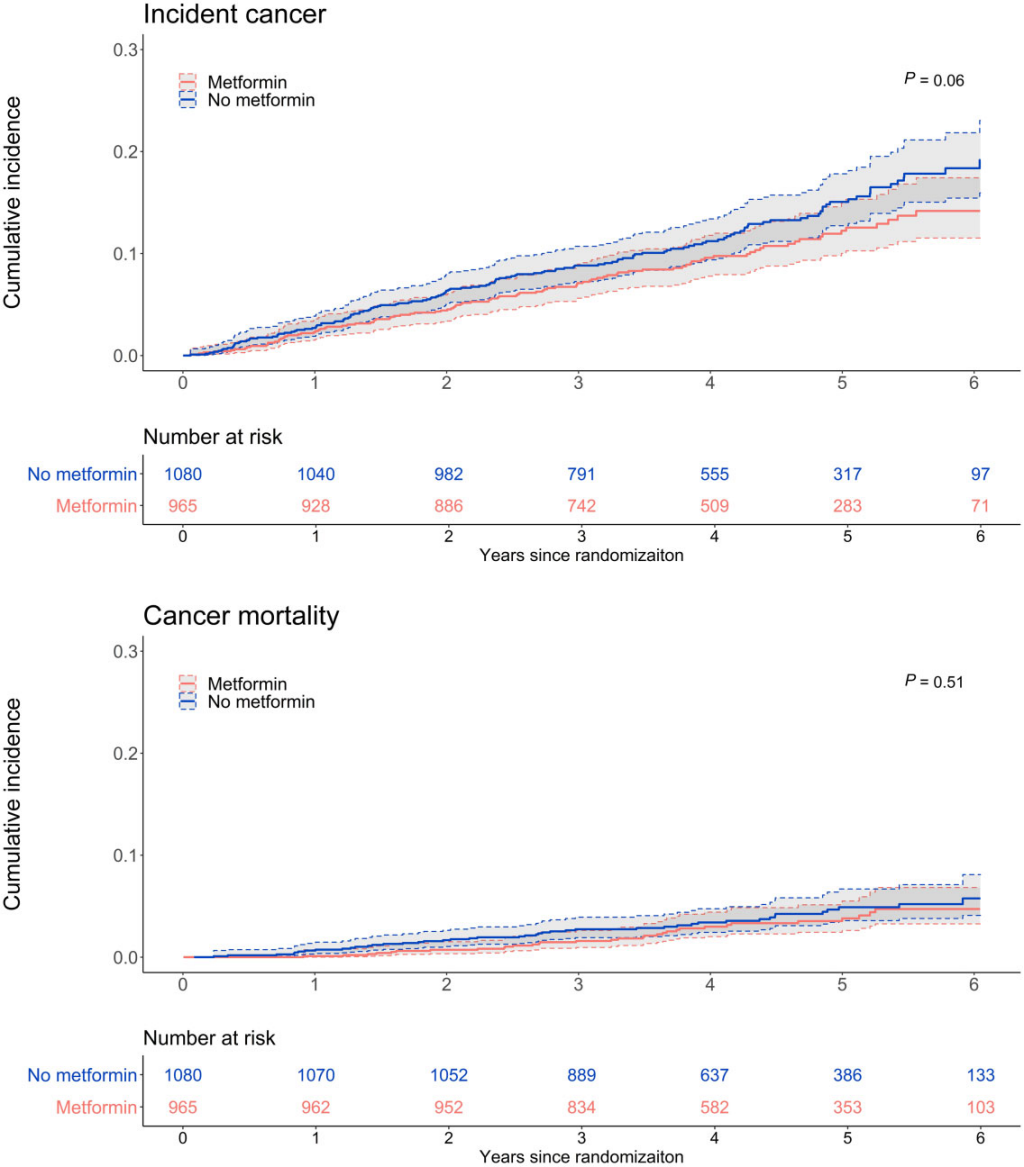
Nelson-Aalen cumulative-incidence curves (95% confidence interval) for cancer incidence and mortality in people with diabetes by metformin use. *P* values (top-left corner) were calculated using Gray tests.

Table 2 describes the relationship between metformin use and cancer incidence and mortality in participants with and without diabetes. After adjustment for baseline characteristics, including FBGL, there was a lower rate of cancer incidence in the metformin group than in the no metformin group (adjusted HR = 0.68, 95% confidence interval [CI] = 0.51 to 0.90), but no significant differences were observed for cancer mortality (adjusted HR = 0.72, 95% CI = 0.43 to 1.19). Metformin users had similar event rates to people without diabetes for cancer incidence (adjusted HR = 1.09, 95% CI = 0.88 to 1.35) and cancer mortality (adjusted HR = 1.39, 95% CI = 0.96 to 2.02), while people with diabetes who did not use metformin had higher rates of cancer incidence (adjusted HR = 1.35, 95% CI = 1.13 to 1.62) and cancer mortality (adjusted HR = 1.55, 95% CI = 1.12 to 2.15). Supplementary analysis with competing-risks regression were consistent with these results (Supplementary Table 3, available online). The cumulative-incidence curves show that metformin users have lower cumulative cancer incidence over time but not lower rates of cancer mortality (Figure 2).

**Table 2.** Relationship between metformin use and cancer incidence and mortality compared with people with diabetes but no metformin use and those without diabetes

	Diabetes			No diabetes, No. (rate)^a^	Adjusted HR (95% CI) diabetes and metformin vs no diabetes^b^	Adjusted HR (95% CI) diabetes and no metformin vs no diabetes^c^
	Metformin, No. (rate)^a^	No metformin, No. (rate)^a^	Adjusted HR (95% CI) metformin vs no metformin^b^			
Incident cancer^d^	101 (25.73)	144 (33.06)	0.68 (0.51 to 0.90)	1688 (22.8)	1.09 (0.88 to 1.35)	1.35 (1.13 to 1.62)
Cancer mortality^e^	34 (7.93)	44 (9.28)	0.72 (0.43 to 1.19)	437 (5.51)	1.39 (0.96 to 2.02)	1.55 (1.12 to 2.15)

a No. = Number of participants with a cancer event. Rate is the event rate per 1000 person-years. BMI = body mass index; CI = confidence interval; CKD = chronic kidney disease; FBGL = fasting blood glucose level; HR = hazard ratio.

b Adjusted for age at randomization, sex, ethnicity (Caucasian/White vs other, where other includes Aboriginal/Torres Strait Islanders, American Indian, Asian, Black/African American, Hispanic/Latino or Native Hawaiian/Other Pacific Islander/Maori), BMI (as continuous), smoking status (current and former vs never), alcohol status (current and former vs never), previous aspirin use, CKD, treatment group, polypharmacy, family cancer history, physical component summary score, personal cancer history, insulin use, other oral antihyperglycemic medication use, and FBGL.

c Adjusted for age at randomization, sex, ethnicity (Caucasian/White vs other), BMI (as continuous), smoking status (current and former vs never), alcohol status (current and former vs never), previous aspirin use, CKD, treatment group, polypharmacy, family cancer history, physical component summary score, and personal cancer history. Baseline characteristics of participants without diabetes are shown in Supplementary Table 2 (available online).

d Time from randomization to first cancer event.

e Time from randomization to cancer-related death. This value includes only deaths that were adjudicated to be cancer related. Cancer deaths where the participant did not present with cancer before death were also included (n = 20).

**Figure 3.**
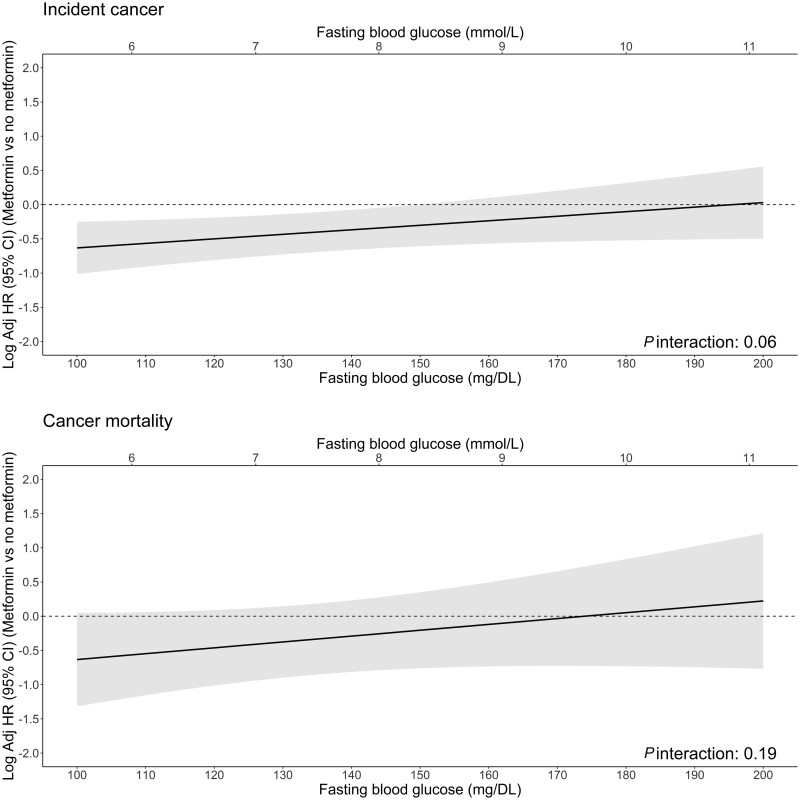
Log-adjusted hazard ratios (HRs) (95% confidence interval [CI]) of metformin (vs no metformin) across varying levels of baseline fasting blood glucose. The log-adjusted hazard ratios were determined by using Cox regression models, with an interaction between metformin and blood glucose, adjusting for baseline confounders: age at randomization, sex, ethnicity (Caucasian/White vs other), body mass index (as continuous), smoking status (current and former vs never), alcohol status (current and former vs never), previous aspirin use, chronic kidney disease, treatment group, polypharmacy, family cancer history, physical component summary score, personal cancer history, insulin use, and other oral antihyperglycemic medication use.

The association of metformin with cancer incidence and mortality as well as continuous FBGL measures are shown in Figure 3. Visually, there was a suggestion that FBGL modified the association between metformin and cancer incidence (interaction effect *P* = .06), suggesting that the benefit of metformin may be more pronounced in those with lower FBGLs.

The combined effect of metformin and aspirin is shown in Table 3. Among those using metformin, those randomized to aspirin had a similar rate of cancer incidence (HR = 1.11, 95% CI = 0.75 to 1.64) but a significantly greater rate of cancer mortality (HR = 2.53, 95% CI = 1.18 to 5.43) compared with placebo. For those without metformin exposure, event rates were similar between participants randomized to aspirin and placebo for cancer incidence (HR = 1.10, 95% CI = 0.79 to 1.52) and mortality (HR = 1.16, 95% CI = 0.64 to 2.09). The possible effect modification on cancer mortality, however, was not statistically significant (interaction *P* = .11). Competing-risks regression supplementary analysis produced similar results (Supplementary Table 4, available online).

**Table 3.** Effect of metformin and aspirin use on cancer incidence and mortality in those with diabetes

	Metformin			No metformin			*P* interaction of metformin and aspirin^a^
	Aspirin	Placebo	HR^a^	Aspirin	Placebo	HR^a^	
	No. (rate)^b^	No. (rate)^b^	(95% CI)	No. (rate)^b^	No. (rate)^b^	(95% CI)	
Incident cancer^c^	56 (26.94)	45 (24.37)	1.11 (0.75 to 1.64)	70 (34.55)	74 (31.76)	1.10 (0.79 to 1.52)	.97
Cancer mortality^d^	25 (10.99)	9 (4.47)	2.53 (1.18 to 5.43)	22 (9.98)	22 (8.67)	1.16 (0.64 to 2.09)	.11

a Unadjusted because treatment allocation to aspirin or placebo was randomized. CI = confidence interval; HR = hazard ratio.

b No. = Number of participants with a cancer event. Rate is the event rate per 1000 person-years.

c Time from randomization to first cancer event.

d Time from randomization to cancer-related death. This value includes only deaths that were adjudicated to be cancer related.

## Discussion

In older people with diabetes, we found that a relationship exists between metformin and cancer prevention that may be modified by lower FBGLs. Overall, we found that people with diabetes whose physician had prescribed metformin had a lower cancer incidence risk than those whose physicians had not prescribed metformin over 4.5 years of follow up. We found no conclusive associations between metformin and cancer mortality, however, likely because of small event numbers. Furthermore, the rate of incident cancer in those on metformin was similar in ASPREE participants who did not have diabetes, indicating that the benefits associated with metformin may potentially attenuate diabetes as a risk factor for cancer.

Several potential explanations exist for the risk reduction associated with metformin use that we observed. A chemoprevention effect of metformin has been attributed to several biological mechanisms, including 1) activation of the liver kinase B-1–adenyl-monophosphate protein kinase pathway and subsequent suppression of hepatic glucose production leading to a reduction in insulin requirements (50,51) and 2) direct effect on cancer cells through reduction in insulin and/or insulin-like growth factor-I (IGFI) receptor signaling (52,53) and inhibition the mammalian target of rapamycin pathway by Adenosine Monophosphate-activated protein kinase–dependent mechanisms reducing adenosine triphosphate synthesis (52,54,55). Therefore, metformin’s mechanism of chemoprevention is not thought to be solely attributable to adequate control of blood glucose but also to its ability to reduce hyperinsulinemia and subsequent insulin signaling pathway activity.

Our data indicated that the beneficial associations of metformin on cancer incidence may not be observed in those with high FBGLs (>150 mg/dL), suggesting that if hyperglycemia and hyperinsulinemia persist, then metformin may have limited clinical effect on cancer risk. Although our analysis of the interaction between FBGL and metformin on cancer incidence was not conclusive, previous studies have shown that blood glucose control is essential for minimizing the risk of microvascular complications, a condition that emerging evidence shows is associated with future risk of cancer (56,57). Our results are also broadly consistent with the American Diabetes Association recommendations for target glucose levels to minimize diabetes-related morbidity (FBGL <150 mg/dL or hemoglobin A_1c_ [HbA_1c_] below equivalent cutoff). Although not conclusive, our results suggest that metformin may make little difference to outcomes if FBGLs are above the American Diabetes Association recommended level.

The ASPREE clinical trial found no difference between aspirin and placebo for cancer incidence but an increased risk of cancer mortality with aspirin (37). In particular, ASPREE demonstrated an increased risk of cancer-related mortality with aspirin regardless of diabetes status, especially for stage III and above cancers (37,38). Given that our analysis used the same data but focused on the subgroup with diabetes, we expected to observe an increased cancer mortality risk with aspirin. Our goal was specifically to explore whether metformin use modified this risk. We found that for metformin users, aspirin use compared with placebo was associated with a significantly increased risk of cancer mortality.

Theoretically, aspirin could increase cancer risk through hyperinsulinemia. Several clinical trials conducted in the 1980s demonstrated a detrimental effect of aspirin therapy on insulin sensitivity in people with (58) and without diabetes (59,60). A more recent clinical study in healthy obese people showed that high-dose aspirin reduced hepatic glucose production and peripheral plasma glucose levels, but these effects were at the expense of a 47% increase in plasma insulin concentrations (61). Therefore, it is plausible that the insulin-attenuating action of metformin may present only in the absence of an aspirin-induced increase in plasma insulin concentration and that aspirin use could result in net harm for cancer outcomes.

Given that the magnitude of the elevated cancer mortality risk observed within the metformin group was pronounced and greater than the hazard ratio observed for the overall cohort (37), we explored whether the risks of aspirin on cancer mortality were modified or indeed magnified with metformin use. Our results do not, however, provide sufficient evidence to draw this conclusion. Although we observed markedly different hazard ratios for the estimated effect of aspirin on cancer mortality within the metformin (HR = 2.53, 95% CI = 1.18 to 5.43) and no metformin groups (HR = 1.16, 95% CI = 0.64 to 2.09), our sample size was limited, and the interaction effect comparing the hazard ratios was low (*P* = .113) but not statistically significant. A relatively small proportion of ASPREE participants had diabetes (10.6%); of these, fewer than half were prescribed metformin, and a smaller proportion still experienced cancer mortality. Thus, although our data showed significantly increased risk of cancer mortality with aspirin among metformin users, we cannot be sure whether the differences in aspirin effects we observed between the metformin and no metformin groups were the result of a true effect modification by metformin or of other factors.

Previous meta-analyses of aspirin clinical trials conducted in middle-aged individuals (ranging in median age at randomization from 57.5 to 66.9 years) found that aspirin treatment prevented cancer, particularly colorectal cancer, over the next 20 years (34,35). The majority of the studies in these meta-analyses, however, were conducted before the introduction of metformin into mainstream use in the United States, which occurred in 1995 (62); as such, they will not have metformin as a confounder. Within the United States today, however, approximately 61.7% of people with diabetes who are older than 60 years of age and likely now taking metformin use aspirin for primary prevention, and this number is increasing with time (63). Taken together, then, much of the evidence supporting aspirin for cancer prevention in middle-aged people was gathered from metformin-naive populations, and much of the recent observational data being used to examine metformin chemoprevention were likely gathered from aspirin-enriched populations. Our results are not conclusive, but we believe that they provide incentive to better understand the relationship among metformin, aspirin, and cancer outcomes, particularly in older individuals with diabetes, through research using larger cohorts and trials.

###  

A key strength of our study was its prospective design, with regular clinical screening and robust clinical event adjudication that minimized ascertainment bias. Our cohort had detailed baseline data collection with limited missing data, including concise ascertainment of medication use (83% of the study population maintained their baseline status of metformin use or nonuse throughout the follow-up period), and we were able to adjust for a wide range of demographic, lifestyle, and known risk factors. Randomization of participants to aspirin or placebo enabled us to analyze the effect of aspirin among metformin users while minimizing confounding bias.

We were limited by the data available to define diabetes, however. Only a single measure of FBGL was collected at enrollment, and HbA_1c_ was not collected. Therefore, diabetes was defined using a single FBGL measure rather than serial FBGLs or HbA_1c_. Consequently, the proportion of people with baseline diabetes may be overestimated. Regardless, the total number of participants with diabetes was limited; hence, event numbers in those with metformin exposure was low. This limitation prevented further statistical testing of the effect of metformin and aspirin on cancer by anatomical location. Additionally, we did not capture pre-enrollment diabetes duration (date of diagnosis) nor commencement date of metformin; thus, we could not address the concept of metformin treatment latency effects.

In community-dwelling older people with diabetes, metformin use was associated with reduced cancer incidence. Aspirin use was associated with increased cancer mortality risk in metformin users, but the modification effect of metformin and aspirin did not reach statistical significance. Further research is required to understand the relationship among metformin, aspirin, and cancer risk.

## Supplementary Material

pkad017_Supplementary_Data

## Data Availability

The data underlying this article cannot be shared because the detail, complexity, and size make them reidentifiable, and privacy of the individuals who participated in the study must be maintained. However, the underlying data can be accessed and analyzed in a secure environment on reasonable request via application through ASPREE.AMS@monash.edu. Applications will be reviewed for scientific merit and successful applicants provided access to participant-level data within a secured data sharing platform. The ASPREE protocol can be publicly accessed via https://aspree.org/usa/wp-content/uploads/sites/3/2021/07/ASPREE-Protocol-Version-9_-Nov2014_FINAL.pdf.
