# Supplementary material for: Association of metformin, aspirin, and cancer incidence with mortality risk in adults with diabetes
Source: JNCI Cancer Spectr. 2023 Mar 1;7(2):pkad017. doi: 10.1093/jncics/pkad017 (PMC10042437; doi:10.1093/jncics/pkad017)
Supplement: pkad017_Supplementary_Data [file pkad017_supplementary_data.zip › SUPPLEMENTARY MATERIAL_SOrchard 12Dec 2022 (1).pdf]

SUPPLEMENTARY MATERIAL

Supplementary Table 1: Antihyperglycemic medication use (ATC code commencing A10A or A10B) in those with diabetes at study enrolment (online only).

Diabetic medications	Metformin (N=965)	No metformin (N=1080)	Total (N=2045)
Acarbose (A10BF01)	3 (0.31%)	1 (0.09%)	4 (0.2%)
Canagliflozin (A10BK02)	2 (0.21%)	0 (0%)	2 (0.1%)
Exenatide (A10BJ01)	10 (1.04%)	2 (0.19%)	12 (0.59%)
Glibenclamide (A10BB01)	40 (4.15%)	15 (1.39%)	55 (2.69%)
Gliclazide (A10BB09)	162 (16.79%)	63 (5.83%)	225 (11%)
Glimepiride (A10BB12)	41 (4.25%)	13 (1.2%)	54 (2.64%)
Glipizide (A10BB07)	31 (3.21%)	21 (1.94%)	52 (2.54%)
Insulin (A10A)	83 (8.6%)	74 (6.85%)	157 (7.68%)
Linagliptin (A10BH05)	6 (0.62%)	3 (0.28%)	9 (0.44%)
Liraglutide (A10BJ02)	1 (0.1%)	0 (0%)	1 (0.05%)
Metformin (A10BA02)	965 (100%)	0 (0%)	965 (47.19%)
Pioglitazone (A10BG03)	32 (3.32%)	15 (1.39%)	47 (2.3%)
Repaglinide (A10BX02)	2 (0.21%)	1 (0.09%)	3 (0.15%)
Rosiglitazone (A10BG02)	6 (0.62%)	1 (0.09%)	7 (0.34%)
Saxagliptin (A10BH03)	10 (1.04%)	5 (0.46%)	15 (0.73%)
Sitagliptin (A10BH01)	60 (6.22%)	25 (2.31%)	85 (4.16%)
Vildagliptin (A10BH02)	14 (1.45%)	0 (0%)	14 (0.68%)

Supplementary Table 2: Baseline characteristics of ASPREE participants without diabetes.

	No diabetes (N=17,069)
Age at randomization (yrs)	
65-69	421 (2%)
70-74	9561 (56%)
75-79	4479 (26%)
80-84	1959 (11%)
85+	649 (4%)
Sex	
Male	7286 (43%)
Ethnicity/Race[a]	
White	15786 (92%)
BMI category	
BMI ≥25kg/m^2	12290 (72%)
Smoking	
Current	637 (4%)
Former	6893 (40%)
Never	9539 (56%)
Alcohol use	
Current	13262 (78%)
Former	948 (6%)
Never	2859 (17%)
Clinical features	
Previous regular aspirin use[b]	1735 (10%)
CKD[c]	4027 (25%)
Polypharmacy (5+)	4023 (24%)
Personal cancer history	3274 (19%)
Family cancer history[d]	10276 (60%)
PCS[e] Median (Interquartile range, IQR)	50.7 (43.4, 55.3)
Randomized Treatment Group	
Aspirin	8501 (50%)
Placebo	8568 (50%)
Fasting Glucose Level (FBGL)	
FBGL in mg/DL Mean (SD)<br>FBGL in mmol/L Mean (SD)	94.9 (10.3)<br>5.3 (0.6)

[a] Ethnicity: White/Caucasian or other (not shown), the latter consists of Hispanic/Latino, Aboriginal/TSI, Native Hawaiian/Other Pacific Islander/Maori, Asian, American Indian, or Black/African American.

[b] Previous regular aspirin use: Self-reported regular aspirin use before entering the study.

[c] Stage 3-5 Chronic kidney disease: Urine albumin creatinine ratio of 3 mg/mmol or more, and/or estimated glomerular filtration rate of less than 60ml per minute per 1.73m^2.

[d] Family cancer history: Cancer history in the participant's blood relatives (mother, father, siblings and children), self-reported at baseline. Ovarian and cervical cancer history were included, which were not included in recent ASPREE publication (26).

[e] Physical component score: physical component score of the SF-12 quality of life questionnaire (61)).

Missing data in total cohort (n = 19,114): age at randomization n=0; sex n=0; ethnicity n=0; BMI n=89, smoking n=0, alcohol use n=0, previous regular aspirin use n=2, CKD n=1350,

polypharmacy n=0, family cancer history n=0, randomized treatment group n=0, physical component score of the SF-12 n=8, personal cancer history n=22.
Percentages exclude missing values from denominator.

Supplementary Table 3: Competing risks regression via Fine-Gray subdistribution hazard models on metformin use and cancer incidence and mortality compared with people with diabetes but no metformin use, and those without diabetes.

	Adj HR§ (95% CI) Metformin vs no metformin[c]	Adj HR (95%CI) Diabetes & Metformin vs No diabetes[d]	Adj HR (95%CI) Diabetes & no metformin vs No diabetes[d]
Incident cancer[a]	0.68 (0.51, 0.91)	1.09 (0.88, 1.35)	1.35 (1.13, 1.61)
Cancer mortality[b]	0.72 (0.43, 1.21)	1.40 (0.96, 2.04)	1.54 (1.11, 2.13)

[a] Death was considered a competing risk of cancer incidence. 466 participants (60 with diabetes) died, while still at risk of cancer incidence, without reaching the cancer incidence endpoint.
[b] Non-cancer related death is defined as a competing risk of cancer mortality. 537 participants (77 with diabetes) died from non-cancer causes (including deaths where cause of death was not yet adjudicated [n = 25]).
[c] Adjusted for age at randomization, sex, ethnicity (Caucasian/white vs other), BMI (as continuous), smoking status (current and former vs never), alcohol status (current and former vs never), previous aspirin use, CKD, treatment arm, polypharmacy, family cancer history, PCS, personal cancer history, insulin use, other oral antihyperglycemic medication use, and fasting blood glucose.
[d] Adjusted for age at randomization, sex, ethnicity (Caucasian/white vs other), BMI (as continuous), smoking status (current and former vs never), alcohol status (current and former vs never), previous aspirin use, CKD, treatment arm, polypharmacy, family cancer history, PCS, and personal cancer history.

Supplementary Table 4: Competing risks regression via Fine-Gray subdistribution hazard models on metformin and aspirin use on cancer incidence and mortality in those with diabetes.

	Metformin	No metformin	*p* Interaction of metformin and aspirin[a]
	Aspirin vs placebo HR (95% CI)	Aspirin vs placebo HR (95% CI)	
Incident cancer	1.11 (0.75, 1.64)	1.09 (0.79, 1.52)	0.97
Cancer mortality	2.52 (1.17, 5.45)	1.15 (0.64, 2.08)	0.12

[a] Unadjusted because treatment arms were randomized.
