# Supplementary material for: Association of metformin, aspirin, and cancer incidence with mortality risk in adults with diabetes
Source: JNCI Cancer Spectr. 2023 Mar 1;7(2):pkad017. doi: 10.1093/jncics/pkad017 (PMC10042437; doi:10.1093/jncics/pkad017)
Supplement: pkad017_Supplementary_Data [file pkad017_supplementary_data.zip › McNeil_2018_-Primary-Supplement._Disability free survival._ASPREE Investigators.docx]

**SUPPLEMENTARY APPENDIX**

This appendix has been provided by the authors to give readers additional information about the following paper:

McNeil JJ, Woods RL, Nelson MR et al for the ASPREE investigators: Effect of aspirin on disability-free survival in the healthy elderly, *N Engl J Med*. DOI: 10.1056/NEJMoa1800722.

1. ASPREE INVESTIGATORS AND COMMITTEES

**ASPREE Grant Investigaotrs:**

John McNeil, Robyn Woods, Rory Wolfe, Anne Murray, Andrew Chan, Suzanne Orchard, Jessica Lockery, Mark Nelson, Christorpher Reid, Raj Shah, Anne Newmann, Elsdon Storey, Nigel Stocks, Andrew Tonkin, Sara Espinoza.

**International Steering Committee**

John McNeil (Chair and Principal Investigator), Anne Murray (Co-Chair and Co-Principal Investigator), Lawrie Beilin, Andrew Chan, Jamehl Demons, Michael Ernst, Sara Espinoza, Matthew Goetz, Colin Johnston, Brenda Kirpach, Danny Liew, Karen Margolis, Frank Meyskens, Mark Nelson, Chris Reid, Raj Shah, Elsdon Storey, Andrew Tonkin, Rory Wolfe, Robyn Woods, John Zalcberg

**International End Point Adjudication Committees**

Mark Nelson (Chair), Diane Ives (Co-Chair), Michael Berk, Wendy Bernstein, Donna Brauer, Christine Burns, Trevor Chong, Geoff Cloud, Jamehl Demons, Geoffrey Donnan, Charles Eaton, Paul Fitzgerald, Peter Gibbs, Andrew Haydon, Michael Jelinek, Finlay Macrae, Suzanne Mahady, Mobin Malik, Karen Margolis, Catriona McLean, Anne Murray, Anne Newman, Luz Rodriguez, Suzanne Satterfield (deceased), Raj Shah, Elsdon Storey, Jeanne Tie, Andrew Tonkin, Gijsberta van Londen, Stephanie Ward, Jeff Williamson, Erica Wood, John Zalcberg

**Data and Safety Monitoring Board**

Jay Mohr (Chair), Garnet Anderson, Stuart Connolly, Larry Friedman, JoAnn Manson, Mary Sano, Sean Morrison, Erik Magnus Ohman

**National Institutes of Health [National Institute on Aging (NIA) and National Cancer Institute (NCI)] oversight**

NIA - Evan Hadley, Judy Hannah, Sergei Romashkan; NCI – Leslie Ford, Ellen Richmond, Asad Umar

**Australian Management Committee**

John McNeil (Chair), Robyn Woods (Deputy Chair), Walter Abhayaratna, Lawrie Beilin, Geoffrey Donnan, Peter Gibbs, Colin Johnston, Danny Liew, Trevor Lockett, Mark Nelson, Chris Reid, Nigel Stocks, Elsdon Storey, Andrew Tonkin, Rory Wolfe, John Zalcberg

**Publications, Presentations and Ancillary Studies Committee**

Anne Murray (Chair), Chris Reid (Co-Chair), Walter Abhayaratna, Michael Ernst, Colin Johnston, Beth Lewis, Danny Liew, Karen Margolis, John McNeil, Mark Nelson, Anne Newman, Thomas Obisesan, Raj Shah, Elsdon Storey, Robyn Woods

**International Data Management Committee**

Chris Reid (Chair), Jessica Lockery (Co-Chair), Michael Ernst, Dave Gilbertson, Brenda Kirpach, Raj Shah, Rory Wolfe, Robyn Woods

**ASPREE Data Management Center (Monash University) and Biostatistics**

Jessica Lockery (Data Manager), Taya Collyer, Jason Rigby; Programmers - Kunnapoj Pruksawongsin, Nino Hay; Biostatisticians – Rory Wolfe (Senior Biostatistician), Joanne Ryan, Kim Jachno, Catherine Smith; End point Processing – A.R.M.Saifuddin Ekram (Clinical Case Reviewer), Madeleine Gardam, Henry Luong, Tim Montgomery, Megan Plate, Laura Rojas, Anna Tominaga, Katrina Wadeson

**Australian Training, Recruitment, Retention and Operations Committee**

Suzanne Orchard (Chair), Sharyn Fitzgerald, Sarah Hopkins, Jessica Lockery, Trisha Nichols, Ruth Trevaks, Robyn Woods

**U.S. Operations/Recruitment and Retention Committee**

Brenda Kirpach (Chair), Ashley Johnson, Anne Murray, Molly Prozinski, Ramona Robinson-O’Brien, Nate Tessum

# SITE PRINCIPAL AND GP ASSOCIATE INVESTIGATORS

**U.S. (2,411 participants):**

John Aloia, Steve Anton, Jeffery Burns, Gary Burton, Jamehl Demons, Charles Eaton, Michael Ernst, Sara Espinoza, Darron Ferris, Mahalakshmi Honasoge, Daniel Hsia, Steven Katzman, Anupama Kottam, Beth Lewis, Karen Margolis, Anne Murray, Shawna Nesbitt, Anne Newman, Thomas Obisesan, Augusto Ochoa, Pricilla Pemu, Kevin Peterson, James Powell, Gregg Pressman, William Robinson III, Susanne Satterfield (deceased), Raj Shah, Christine Thorburn, Elena Volpi, Jocelyn Wiggins, Jeff Williamson, Peter Wilson, Catherine Womack
